# Growth and physiological responses of three warm-season legumes to water stress

**DOI:** 10.1038/s41598-020-69209-2

**Published:** 2020-07-22

**Authors:** Gurjinder S. Baath, Alexandre C. Rocateli, Vijaya Gopal Kakani, Hardeep Singh, Brian K. Northup, Prasanna H. Gowda, Jhansy R. Katta

**Affiliations:** 1 Department of Plant and Soil Sciences, Oklahoma State University, 371 Agricultural Hall, Stillwater, OK 74078 USA; 2 USDA-ARS, Grazinglands Research Laboratory, 7207 W. Cheyenne St., El Reno, OK 73036 USA; 3 USDA-ARS, Southeast Area, 114 Experiment Station Road, Stoneville, MS 38776 USA

**Keywords:** Plant physiology, Abiotic, Drought, Natural variation in plants

## Abstract

Novel drought-tolerant grain legumes like mothbean (*Vigna acontifolia*), tepary bean (*Phaseolus acutifolius*), and guar (*Cyamopsis tetragonoloba*) may also serve as summer forages, and add resilience to agricultural systems in the Southern Great Plains (SGP). However, limited information on the comparative response of these species to different water regimes prevents identification of the most reliable option. This study was conducted to compare mothbean, tepary bean and guar for their vegetative growth and physiological responses to four different water regimes: 100% (control), and 75%, 50% and 25% of control, applied from 27 to 77 days after planting (DAP). Tepary bean showed the lowest stomatal conductance (*g*_s_) and photosynthetic rate (*A*), but also maintained the highest instantaneous water use efficiency (*WUE*_*i*_) among species at 0.06 and 0.042 m^3^ m^−3^ soil moisture levels. Despite maintaining higher *A*, rates of vegetative growth by guar and mothbean were lower than tepary bean due to their limited leaf sink activity. At final harvest (77 DAP), biomass yield of tepary bean was 38–60% and 41–56% greater than guar and mothbean, respectively, across water deficits. Tepary bean was the most drought-tolerant legume under greenhouse conditions, and hence future research should focus on evaluating this species in extensive production settings.

## Introduction

Legume crops are an integral component of many cropping systems, and provide multiple ecosystem services essential for agricultural sustainability. Besides delivering pulses (grains) for humans and forage for livestock, legumes add organic nitrogen to the soil, provide cover to reduce runoff and soil erosion, mitigate greenhouse gas emissions, and increase carbon sequestration^1,2^. The continuous rise in prices for inorganic nitrogen fertilizers has stimulated growers in many countries, including the United States (US), to incorporate legumes as grain, forage, or green cover into different cropping systems^3,4^. Although legumes utilized as either forage or green cover result in lower biomass yields than cereals, they provide livestock with herbage of greater N concentrations and digestibility, which are important for growing animals^5^.

Forage-livestock production systems used in the US Southern Great Plains (SGP) largely depend on winter wheat (*Triticum aestivum* L.) during fall through spring, and on perennial grasses such as old world bluestems (*Bothriochloa* spp.), bermudagrass [*Cynodon dactylon* (L.) Pers.], or native prairie during summer for grazing stocker cattle^6,7^. However, available forage provided by these perennial grasses often show declines in their yield rates and nutritive values by mid-summer, which can limit the rate of weight gain in stockers if not provided expensive protein diets supplements^8,9^. Therefore, the potential of less common, novel grain legumes in providing ample amounts of high-quality forage while also increasing nitrogen levels in soils need to be investigated to improve the sustainability of forage-stocker production systems.

Research conducted in the SGP over the last two decades has focused on defining the forage and green cover potential of several grain legumes^10–14^. Soybean is the most-commonly legume grown in the region and has been utilized for multiple purpose. Included is use as a summer forage^15^, though soybean generally produces insufficient forage yields (< 1.5 Mg ha^−1^), especially when low rainfall occurs during the early summers^11,14^. In contrast, some of the tested drought-tolerant legumes, such as pigeon pea [*Cajanus cajan* (L.) Millsp.], could provide 5 Mg ha^−1^ dry matter with N concentration greater than 20 g kg^−1^ by late summer^10^. However, the value of many pulses for grazing may also be limited due to the presence of larger, less-digestible stems (435–445 g kg^−1^) in aboveground biomass^10,12,13^, and the presence of condensed tannins in plant tissues, that inhibit grazing^16^. Such issues, in addition to effects on soil water and other resources important to the productivity of winter wheat have resulted in a continuing exploration of the pulses of the world to identify novel species that may function as high-quality forage.

Recent research has identified two novel pulses with potential to serve as forage: tepary bean [*Phaseoulus acutifolius* (A.) Gray]; and mothbean [*Vigna aconitifolia* (Jacq.) Marechal]. Both pulses drew attention due to herbage with high N concentration and fiber digestibility, and plant canopies with finer stems than more-common legumes^17–19^. Further, guar [*Cyamopsis tetragonoloba* (L.) Taub.], a true multi-purpose pulse with industrial uses^20^, also has shown the capacity to produce digestible, high-N biomass when harvested during the vegetative growth phase^13^. A recent field study in SGP reported more digestible stems of tepary bean (661 g kg^−1^) and guar (619 g kg^−1^) compared to soybean (516 g kg^−1^)^21^. In another study, mothbean resulted in forage biomass with an average of 21 g kg^−1^ N and 797 g kg^−1^ in vitro digestibility at maturity, which exceeded values reported for other legumes tested in the region^17^. Therefore, tepary bean, guar and mothbean possess capabilities to generate biomass of an improved nutritive value to support stocker cattle production in the SGP.

The selection of alternate crop species for summer periods in the SGP is critical, as agricultural production is largely rainfed, and the agro-climatic conditions of the region are highly variable. The region often experiences prolonged droughts, and the amount of summer precipitation received is highly erratic on a yearly basis^22,23^. Therefore, it is essential to identify drought-tolerant crops capable of producing significant yields, with minimal amounts of soil moisture, under the variable climatic conditions of the SGP. Tepary bean, mothbean, and guar are known for drought tolerance in their native regions^18^ and have the potential to serve as summer crops in rotation with winter wheat in the SGP. However, an understanding of the comparative response of their vegetative growth to a range of water regimes is essential to determine their adaptability to the SGP.

Reduced transpiration rates (*E*) under stomatal regulation is a typical response of most plants to water stress, which allows them to increase their water use efficiency (WUE). Some species tolerate water stress better than others by partially closing their stomata at higher water potentials, and hence become more efficient at utilizing available water under drought conditions^24^. However, the reduction in stomatal conductance (*g*_*s*_) under severe water stress can cause an imbalance between electron transport required for photosynthesis (*A*) and photochemical activity in photosystem II, and thus lead to photosynthetic inhibition^25^. As growth rates of plants are generally determined by rates of photosynthesis (*A*), an association of higher *A* and improved WUE may result in yield enhancement under conditions of water stress^26^. Therefore, a comparison of physiological responses is necessary to understand how these crop species deal with different levels of drought stress. The objectives of this study were to; (1) compare mothbean, tepary bean and guar for their vegetative growth responses to four water regimes, and (2) analyze the effects of water stress on their physiological processes.

## Results

### Environmental conditions

**Figure 1:**
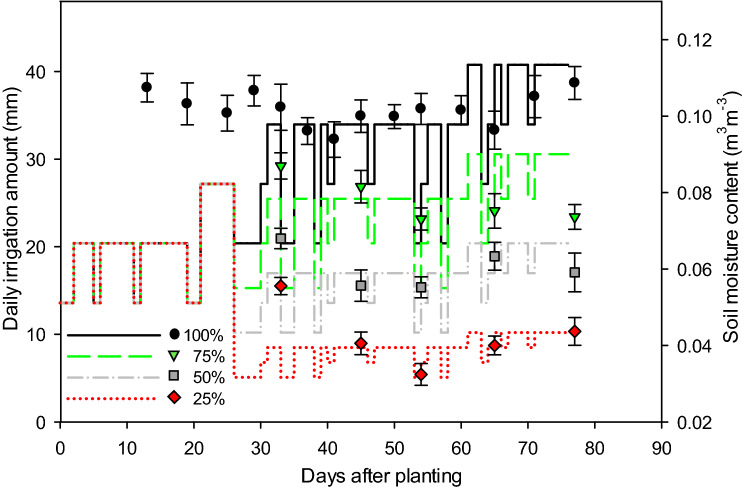
Amounts of daily irrigation applied (lines) and average amounts of soil moisture (points) observed in four water treatments during the growing season. Error bars represent standard error of means (n = 9).

During the experiment period, the average day and night temperatures observed were 30.2 ± 7.8 and 21.3 ± 5.1 °C, respectively, and average humidity was 58.6 ± 6.7%. Amounts of soil moisture at 0–15 cm was monitored regularly in 100% (control) treatment and served as the reference for maintaining three treatments that generated different water deficits (Fig. 1). Measured soil moisture differed substantially among the four water treatments, though no differences were observed among the three species. The average soil moisture (n = 9) of the three species recorded in 100%, 75%, 50% and 25% water levels were 0.101, 0.078, 0.06 and 0.042 m^3^ m^−3^, respectively. Hereafter, the average amount of soil moisture content recorded in four treatments were used for analysis and interpretation of results.

### Physiological responses

**Figure 2:**
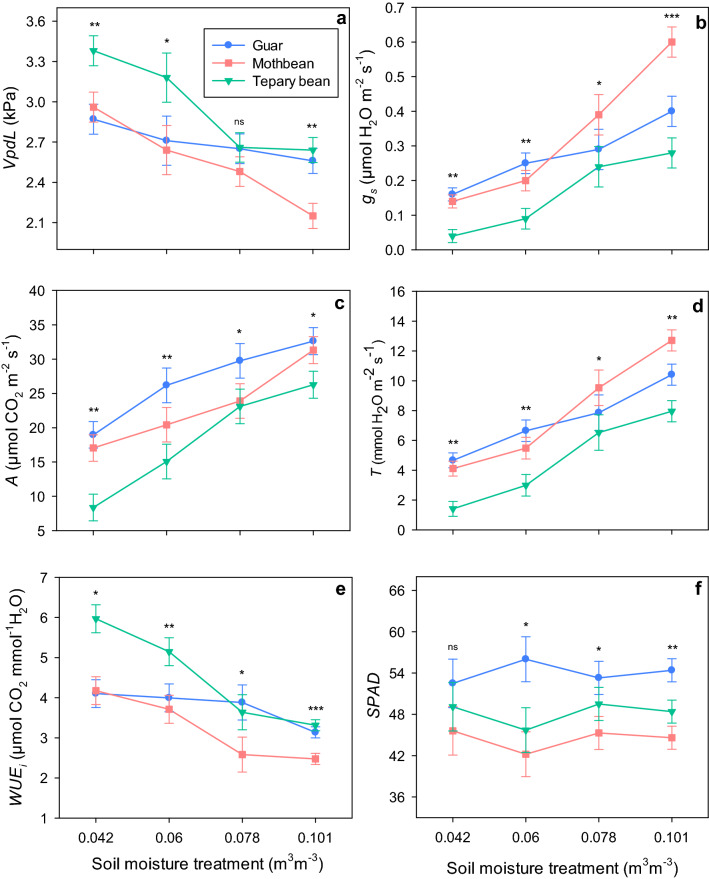
Effect of four levels of water deficit on (**a**) leaf-to-air vapor pressure deficit (*VpdL*), (**b**) stomatal conductance (*g*_s_), (**c**) net photosynthetic rate (*A*), (**d**) transpiration rate (*T*), (**e**) instantaneous water use efficiency (*WUE*_*i*_), and (**f**) SPAD chlorophyll meter reading (*SPAD*) in three warm-season legume species. Error bars are standard errors (n = 4) derived from one-way analysis of variance. Asterisks denote statistically significant differences among species (*p ≤ .05; **p ≤  .01; ***p ≤ .001), and ns indicates non-significant differencess at *p* > .05.

All three species showed typical increase in leaf-to-air vapor pressure deficit (*VpdL*) with increasing amounts of water deficit (Fig. 2a), but differed in intensity of their individual responses (*p* ≤ 0.05) across the four water treatments. Among species, tepary bean experienced a greater *VpdL* of 3.40 kPa and 3.16 kPa at 0.042 m^3^ m^−3^ and 0.06 m^3^ m^−3^ soil moisture, respectively, compared to both guar and mothbean. A similar range of *VpdL* (2.48–2.66 kPa) was observed for all three species at 0.078 m^3^ m^−3^, while mothbean encountered a comparatively lower *VpdL* under the well-watered treatment. The *g*_s_ responses appeared inversely proportional to observed *VpdL* in all three legume species (Fig. 2b). Tepary bean had the lowest observed *g*_s_ under all levels of water deficit, though it was not significantly different (*p* > 0.05) from guar at 0.078 m^3^ m^−3^. Mothbean had the greatest *g*_s_ at soil moisture 0.101 m^3^ m^−3^, but showed responses that were similar to guar under the other water treatments.

The rates of *A* and *T* of all three species declined substantially as water deficit increased (Fig. 2c,d). The decline in *T* occurred relative to *g*_s_ responses across species, while *A* was not entirely proportionate to *g*_s_. The greatest *A* was exhibited by guar under the different soil moisture levels, but was not significantly different (*p* > 0.05) from mothbean at 0.042 and 0.101 m^3^ m^−3^. Alternatively, tepary bean showed the lowest *A* among species across all four levels of water deficit, though a comparable response was observed for mothbean at 0.078 m^3^ m^−3^.

Tepary bean exhibited 43% and 45% higher instantaneous water use efficiency (*WUE*_*i*_) than mothbean and guar, respectively, under the most-severe water stress (0.042 m^3^ m^−3^; Fig. 2e). Likewise, at 0.06 m^3^ m^−3^ soil moisture, it was 39% and 29% higher than mothbean and guar, respectively. In contrast to tepary bean and mothbean, guar did not show larger reductions in *WUE*_*i*_ with increasing water availability. Thus, the *WUE*_*i*_ of guar was consistent and comparable to tepary bean, and higher than mothbean under 0.078 and 0.101 m^3^ m^−3^. No significant effect of soil moisture deficit was observed on *SPAD* values in any of three species (Fig. 2f). Tepary bean and mothbean showed a similar *SPAD* range of 42.2–49.5 across water deficit levels, except a slightly lower value observed for mothbean at 0.101 m^3^ m^−3^. While, a greater *SPAD* range (52.5–56.0) was noted for guar compared to mothbean and tepary bean, though it was not significantly different from tepary bean at 0.042 and 0.078 m^3^ m^−3^ soil moisture.

**Figure 3:**
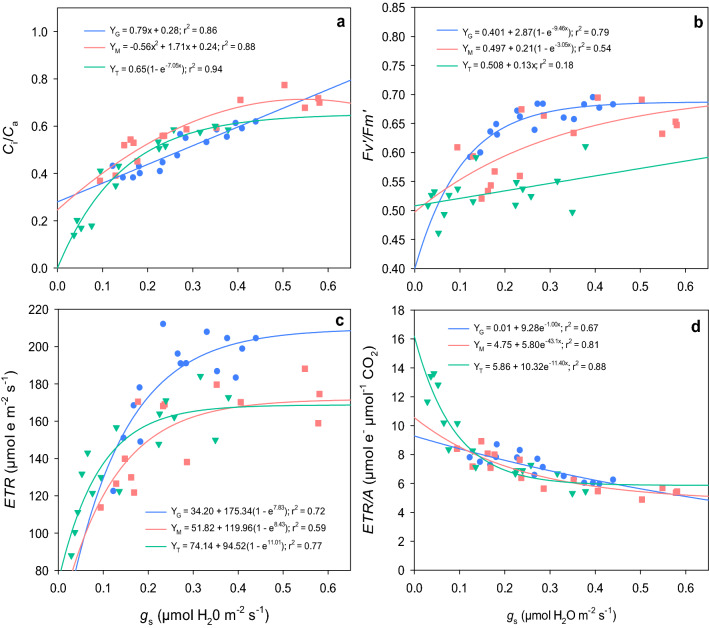
Relationships between stomatal conductance (*g*_s_) and (**a**) *C*_i_/*C*_a_ ratio, (**b**) fluorescence (${{F_{v}^{{\prime }} } / {F_{m}^{{\prime }} }}$), (**c**) electron transport rate (*ETR*) and (**d**) *ETR/A* ratio for three warm-season legume species. Green triangles represent data from tepary bean (T), and pink squares and blue circles represent data from mothbean (M) and guar (G), respectively.

Different responses of ratio of intercellular to ambient CO_2_ concentrations (*C*_i_/*C*_a_) were observed with reducing *g*_s_ in all species, though all declined and none showed increases at the lowest observed values of *g*_s_ (Fig. 3a). A linear relationship of *C*_i_/*C*_a_ with *g*_s_ was obtained for guar (r^2^ = 0.86), while mothbean and tepary bean showed a quadratic (r^2^ = 0.88) and exponential (r^2^ = 0.94) responses, respectively. In comparison to their *C*_i_/*C*_a_ responses, both guar and mothbean showed an exponential decline in ${{F_{v}^{{\prime }} } / {F_{m}^{{\prime }} }}$ with reduction in *g*_s_, with guar showing a steeper slope for the relationship (Fig. 3b). No significant change (*p* > 0.05) in ${{F_{v}^{{\prime }} } / {F_{m}^{{\prime }} }}$ was recorded for tepary bean in response to stomatal closure, although ${{F_{v}^{{\prime }} } / {F_{m}^{{\prime }} }}$ values were generally lower, compared to mothbean and guar.

All three species depicted an exponential decline in electron transport rate (*ETR*) with decrease in *g*_s_ under water stress (Fig. 3c). The *ETR* in guar remained comparatively high under well-watered or mild water stresses (0.22 < *g*_s_ < 0.45) compared to tepary bean and mothbean, but was comparable to both under moderate to severe water stress. Tepary bean maintained a slightly higher *ETR* than the other pulses at *g*_s_ ranging between 0.0 and 0.1. The *ETR/A* value increased in a similar fashion for all three species with decline in *g*_s_ until 0.1 µmol H_2_O m^−2^ s^−1^ (Fig. 3d). Once *g*_s_ was below 0.1 µmol H_2_O m^−2^ s^−1^, tepary bean had a significantly higher *ETR/A* compared to mothbean and guar.

### Vegetative growth responses

**Figure 4:**
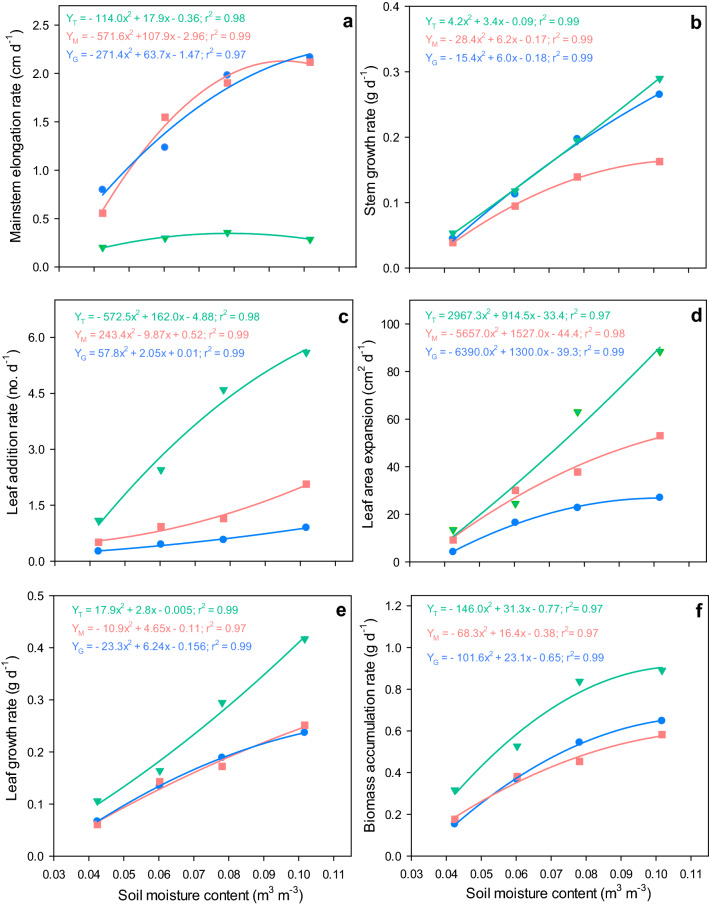
Influence of soil water deficit on (**a**) mainstem elongation rate, (**b**) stem growth rate, (**c**) leaf addition rate, (**d**) leaf expansion rate, (**e**) leaf growth rate, and (**f**) biomass accumulation rate of three warm-season legumes. Green triangles represent data from tepary bean (T), and pink squares and blue circles represent data from mothbean (M) and guar (G), respectively.

Rates of mainstem elongation were influenced by water stress in guar and mothbean, but only minimally effected in tepary bean (Fig. 4a). Both guar and mothbean showed an increase in the rate of mainstem elongation with increasing available soil water, with greatest rates (~ 2.1 cm d^−1^) observed at 0.101 m^3^ m^−3^. Further, similar rates of mainstem elongation (1.9–2.0 cm d^−1^) were exhibited by mothbean and guar at 0.078 m^3^ m^−3^, while responses varied at 0.042 and 0.06 m^3^ m^−3^. Guar had a higher rate of mainstem elongation at 0.042 m^3^ m^−3^, but was surpassed by mothbean at 0.06 m^3^ m^−3^ soil moisture. In comparison, tepary bean showed low rates of mainstem elongation under all soil moisture levels.

The growth rates of stems for all species did not follow trends that were similar to rates of mainstem elongation (Fig. 4b). All three species depicted approximate stem growth rates (0.04–0.05 g d^−1^) at 0.042 m^3^ m^−3^. However, mothbean accumulated lower amounts of stem at 0.06, 0.078, and 0.101 m^3^ m^−3^ than guar or tepary bean. Rates of stem growth in both guar and tepary bean showed a similar increase with increasing water availability, with a slightly higher rate of stem growth for tepary bean under the water watered treatment (0.101 m^3^ m^−3^).

Rates of leaf addition for all three legumes increased substantially as water availability increased (Fig. 4c). Tepary bean showed the greatest rates of leaf addition, while guar showed the lowest rates under all applied treatments. Rates of leaf addition by tepary bean (5.6 leaves d^−1^) were substantially higher than mothbean (2.1 leaves d^−1^), and guar (0.9 leaves d^−1^) at 0.101 m^3^ m^−3^ (control) soil moisture. At 0.042 m^3^ m^−3^, the rate of leaf addition for tepary bean (1.1 leaves d^−1^) was 115% and 313% higher than in mothbean and guar, respectively.

The rate of expansion of leaf area in mothbean and tepary bean were comparable at 0.042 and 0.06 m^3^ m^−3^ soil moisture levels (Fig. 4d). While in congruence with observed rates of leaf addition, tepary bean showed the greatest rates of leaf expansion at 0.078 and 0.101 m^3^ m^−3^ (88.4 cm^2^ d^−1^ and 63.0 cm^2^ d^−1^, respectively). The lowest rates of expansion of leaf area among species were observed for guar, which ranged between 22.7 cm^2^ d^−1^ at 0.042 m^3^ m^−3^ and 26.9 cm^2^ d^−1^ at 0.101 m^3^ m^−3^ moisture level.

The growth rate of leaves increased with increasing amounts of available water for all three species, though tepary bean had greater responses (Fig. 4e). Mothbean and guar showed comparable rates of leaf growth, varying from 0.06 g d^−1^ at 0.042 m^3^ m^−3^ to 0.25 g d^−1^ at 0.101 m^3^ m^−3^. In contrast, leaf growth of tepary bean was steeper, with rates of 0.29 and 0.41 g leaves d^−1^ observed at 0.078 and 0.101 m^3^ m^−3^, respectively.

As with rates of growth by leaves and stems, the rate of accumulation of aboveground biomass was consistently higher for tepary bean compared to mothbean and guar under all levels of water deficit (Fig. 4f). Tepary bean resulted in the greatest rate of biomass accumulation (0.89 g d^−1^ at 0.101 m^3^ m^−3^), which was 37% and 53% higher than rates for mothbean and guar, respectively. Tepary bean also produced biomass at rates that were 80% and 107% higher than mothbean and guar, respectively, under the 0.042 m^3^ m^−3^ treatment.

### Final harvest

**Figure 5:**
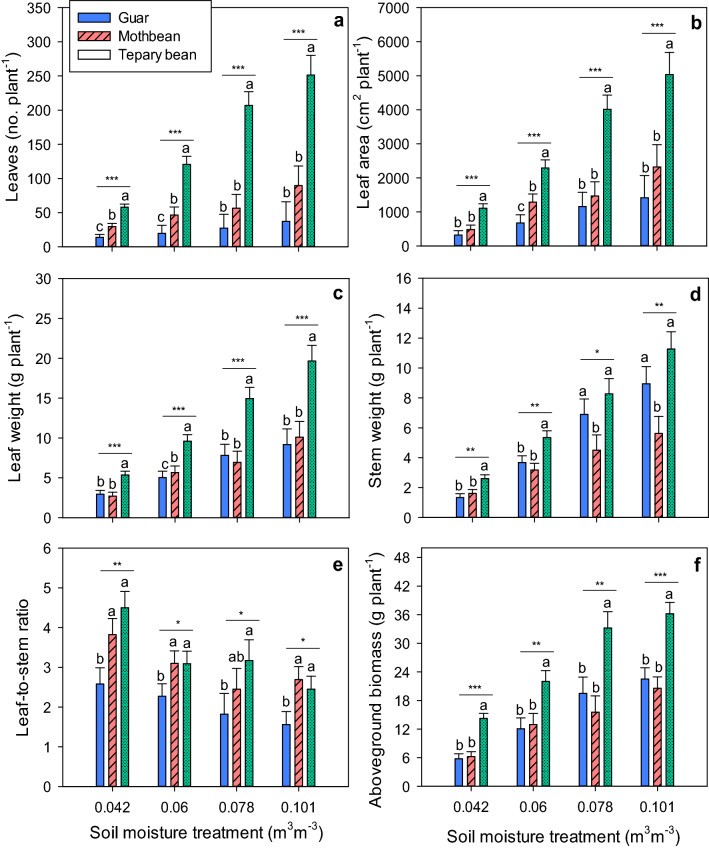
Effects of different water levels on (**a**) number of leaves, (**b**) leaf area, (**c**) leaf weight, (**d**) stem weight, (**e**) leaf-to-stem ratio, and (**f**) aboveground biomass of tepary bean (T), mothbean (M), and guar (G) at final harvest. Bars with same letters within a group of means are not significantly different according to least significant difference (LSD) test at *p* ≤ 0.05. Asterisks denote statistically significant differences among species (*p ≤ .05; **p  ≤ .01; ***p  ≤ .001).

The total number of leaves per plant formed by the three legume species differed significantly (*p* ≤ 0.05) when compared across the four water treatments at 77 days after planting (DAP) (Fig. 5a). Tepary bean had 251.2 leaves plant^−1^ at 0.101 m^3^ m^−3^ soil moisture conditions, which was distinctly higher than guar (37.2 leaves plant^−1^), and mothbean (89.5 leaves plant^−1^). Additionally, tepary bean largely exceeded guar and mothbean in the number of leaves at 0.078, 0.06, 0.042 m^3^ m^−3^. Guar had the least number of leaves per plant under all water treatments, though it was not statistically different from mothbean at 0.0785 and 0.101 m^3^ m^−3^ treatments. Similar results were observed among the species for leaf area under each water treatment (Fig. 5b). Tepary bean resulted in 1.8–2.7 and 3.5 times more leaf area than mothbean and guar, respectively, across the different water levels. Among species, guar showed the lowest leaf area per plant, which ranged between 319 cm^2^ at 0.042 m^3^ m^−3^ and 1,416 cm^2^ at 0.101 m^3^ m^−3^ soil moisture. Although mothbean had comparatively higher leaf area than guar at all water deficit treatments, a statistical difference between mothbean and guar was only observed at 0.06 m^3^ m^−3^.

Significant differences were observed among the legume species for leaf weight per plant in every water treatment (Fig. 5c). Among species, tepary bean had the greatest leaf weights in all four water deficit levels. Guar and mothbean showed similar leaf weights; and both generated half the leaf weight of tepary bean for all levels of water deficit. Alternatively, stem weight per plant did not follow the same trend as leaf weight per plant (Fig. 5d). Mothbean generated the lowest stem weights across all four water treatments, but was similar to responses by guar at 0.042 and 0.06 m^3^ m^−3^. However, the leaf-to-stem ratios obtained for tepary bean were higher and ranged between 2.5 and 4.5 at 0.101 and 0.042 m^3^ m^−3^ soil moisture, respectively (Fig. 5e). The leaf-to-stem ratio of mothbean was comparable to tepary bean, while guar generated the smallest leaf-to-stem ratios in response to each of the four water treatments.

The amounts of aboveground biomass generated by tepary bean were significantly higher than for guar and mothbean across all four water levels (Fig. 5f). Tepary bean produced 38–60% and 41–56% greater biomass yields than guar and mothbean, respectively, under the different water deficits used in the study. In comparison to the potential biomass yield observed under 0.101 m^3^ m^−3^ (control), tepary bean showed a reduction of only 8% at 75% treatment, while guar and mothbean were reduced by 24% and 13%, respectively. Similarly, under severe water deficit (0.042 m^3^ m^−3^), the amount of biomass produced by tepary bean was reduced 60%, compared to 70% and 74% reductions for mothbean and guar, respectively.

## Discussion

Since *VpdL* has an inverse relationship with leaf water potential or relative leaf water content, a higher *VpdL* suggests an insufficient supply of water to leaves by the plant vascular system under condition of water stress^27,28^. Likewise, all three species experienced a range of water deprivation under water deficits, as explained by the observed *VpdL* responses in this study. The variation in magnitude of *VpdL* among species could be related to their different whole-plant hydraulic strategies that occurred under water stress^29^. However, *VpdL* and *g*_s_ showed comparable patterns under the four water treatments, which implied that *VpdL* was an important factor affecting the stomatal functioning in the tested species. Such stomatal behaviors in response to increasing *VpdL* were also earlier reported in other plant species^30,31^.

Due to the sensitivity of *g*_s_ to most of internal and external factors, including *VpdL*, linked to drought, it serves as an integrative basis to understand the influence of water stress on photosynthetic parameters^25^. The stomatal regulated reductions in *A* and *T* vary within species and thus allow some plant species to better tolerate water stress^32^. Similarly, the three legumes evaluated differed in their stomatal behavior, which resulted in different growth and production responses on exposure to the range of water regimes used in this study. Declines in *g*_s_ occurred in each of the three species with increasing water stress, but tepary bean showed greater *g*_s_ reductions than mothbean and guar under moderate (0.06 m^3^ m^−3^) and severe (0.042 m^3^ m^−3^) water treatments. Earlier studies also reported a greater stomatal closure in tepary bean compared to common bean (*Phaseolus vulgaris* L.) across different levels of water deficit^24^. Therefore, the amount of water losses through *T* was better controlled by tepary bean than either mothbean or guar under water-stressed conditions (0.042 and 0.06 m^3^ m^−3^).

Although tepary bean showed a greater decline in *A* due to lesser *g*_s_, *WUE*_*i*_ was substantially higher than either guar or mothbean. In contrast, the *g*_s_ of mothbean was significantly higher under mild (0.078 m^3^ m^−3^) water-stressed and well-watered (0.101 m^3^ m^−3^) conditions. Consequently, mothbean was the least water efficient among the three species due to its greater loss of water through *T* and only moderate increases in *A*. While the increase in *A* was proportional to increasing *T* in guar under the three water levels of deficit, there was little change in instantaneous *WUE*_*i*_.

Other than *A* limitations caused by reductions in *g*_s_, non-stomatal limitations could also occur due to photodamage of PSII from over-excitation under severe conditions of water stress^33^. The occurrence of non-stomatal limitation is generally related to an increase in *C*_i_/*C*_a_ at low *g*_s_ under severe water-stressed conditions^34,35^. While a low *g*_s_ was only observed in tepary bean in this study, it did not cause increases in *C*_i_/*C*_a_ under the severe water-stressed treatment (0.042 m^3^ m^−3^). Since WUE is inversely related to the *C*_i_/*C*_a_ ratio, the lower values of *C*_i_/*C*_a_ obtained with tepary bean at *g*_s_ < 0.1 also revealed an ability to maintain higher WUE compared to guar and tepary bean under severe water stress^25^. Additionally, no significant change in ${{F_{v}^{{\prime }} } / {F_{m}^{{\prime }} }}$ and a higher *ETR* were observed, which suggested that the PSII system of tepary bean was less susceptible to photo-damage under severe water stress, compared to the other tested species.

Although all three species down-regulated their photosynthetic *ETR*, and an increase in *ETR/A* was observed with increasing water stress, a large difference in consumption of electrons for CO_2_ fixation was observed in tepary bean under severe water stress. This implies increased amounts of activity by alternative electron sinks, such as photorespiration, to handle excess electrons generated by photosynthesis^36^; however, it also suggests the potential risk of oxidative damage by reactive oxygen species (ROS) in tepary bean^37^. If oxidative stress occurs, chlorophyll biosynthesis could be impacted and hence photosynthetic activity in plants^38^. However, the plants in this study might not have encountered any oxidative stress, since there was no reduction observed in SPAD chlorophyll readings with increasing water stress for all three species.

A higher ${{F_{v}^{{\prime }} } / {F_{m}^{{\prime }} }}$, and maintenance of *ETR* in guar indicates a greater photochemical efficiency under mild water stress or well-watered conditions compared to mothbean and tepary bean. However, guar showed a rapid decline in ${{F_{v}^{{\prime }} } / {F_{m}^{{\prime }} }}$ and *ETR* at increased water stress, which suggested the downregulation of PSII activity with photo-protective mechanisms such as non-photochemical quenching or increase in thermal energy dissipation^39^. Likewise, the PSII activity was downregulated in mothbean with decrease in *g*_s_, though the responses of ${{F_{v}^{{\prime }} } / {F_{m}^{{\prime }} }}$ and *ETR* remained lower compared to guar. Nevertheless, there was no evidence of non-stomatal limitation, and *A* inhibition could be assumed mainly due to stomatal regulation in all three species.

Rates of plant growth and carbon assimilation are intimately associated, but the extent to which *A* increases plant growth depends on the activity of sinks^40^. In many cases, water stress was found to uncouple *A* and plant growth as carbon assimilation is maintained while sink activity is affected^41^. Similarly, guar and mothbean maintained higher *A*, while their growth rates were lower due to limited leaf sink activity compared to tepary bean under the two treatments that caused the greater water-stress (0.42 and 0.06 m^3^ m^−3^). In contrast, tepary bean maintained a higher rate of leaf addition despite lower *A*, and resulted in greater biomass accumulation than guar and mothbean across all levels of water stress. Additionally, greater biomass production in tepary bean could also be attributed to its vigor at early stages of growth and greater *WUE*_*i*_ under water-stressed conditions^42,43^.

Tepary bean demonstrated lower rates of mainstem elongation compared to guar and mothbean across all water levels, which was mainly due to its vining, more spreading, growth habit, consisting of numerous secondary and tertiary stems^44^. Accordingly, the rate of stem growth and final weight of stem produced by tepary bean were similar, or greater, compared to guar in spite of lower rates of elongation by mainstems. However, the rate of leaf growth and final weight of leaves for tepary bean were greater than guar or mothbean, which can be related to its greater rates of leaf addition and leaf area expansion. Thus, tepary bean not only resulted in greater biomass production but also greater leaf-to-stem ratio than guar across all water treatments, an important feature for forage production to support growing cattle^10,12,13,15,45^.

In terms of forage use, leaves of legumes are generally highly nutritious relative to stems, which contain greater amounts of different fractions of cell wall^45^. Similar findings were also observed for tested species. At 80 DAP, leaves of both tepary bean and guar were noted to contain 32.6 ± 0.7 g kg^−1^ N and 827 ± 12 g kg^−1^ digestibility; while their stems contained 22.8 ± 0.2 g kg^−1^ N and 640 ± 19 g kg^−1^ digestibility, respectively. Likewise, mothbean leaves possessed 26.4 ± 0.3 g kg^−1^ N and 801 ± 16 g kg^−1^ digestibility as compared to its stems with 15.2 ± 0.2 g kg^−1^ N and 671 ± 8 g kg^−1^ digestibility under similar conditions (G. Baath, unpublished data, 2019). Therefore, legume species with high leaf-to-stem ratios could be assumed to enhance forage intake by animal due to a greater digestibility of biomass, and rate of passage through rumen^12,13^. The leaf-to-stem ratios ranging between 2.5 and 4.5 observed for both tepary bean and mothbean indicated their potential as sources of forage with superior nutritive value compared to other well-known legumes^10,13,45^. In comparison, the larger proportion of inferior quality stems generated by guar would limit its value for grazing^12,13,45^. Furthermore, tepary bean possessed greater rates of leaf area expansion and resulted in greater amounts of leaf area per plant at a faster rate than either guar or mothbean across all water regimes. Thus, the capability of tepary bean to generate high amounts of leaf biomass indicates this species may have greater value as a green cover or forage crop than guar or mothbean.

## Conclusion

Stomatal conductance was the main limitation to *A* in all three species under water deficit, and there was no evidence of non-stomatal limitations in the current study. Tepary bean tolerated the applied water stresses better than mothbean or guar due to its greater stomatal closure and *WUE*_*i*_, coupled with more vigorous growth and leaf sink activity during early stages of development. Consequently, tepary bean possessed the highest growth rate and generated the greatest aboveground biomass across all water levels. Growth rates observed for mothbean and guar were similar, though a comparatively greater leaf-to-stem ratio was obtained in mothbean. High leaf-to-stem ratio noticed in both tepary bean and mothbean suggested their potential to function as sources of nutritious forage compared to guar. Further, the greater leaf area noted for tepary bean observed across all water deficits indicates it may also have value as a cover crop capable of reducing soil erosion, suppressing weeds, and providing organic N. Overall, this greenhouse study provided evidence that tepary bean could serve as a reliable choice for further investigation in SGP conditions among the tested species, considering its greater biomass production, *WUE*_*i*_, leaf-to-stem ratio, and soil covering ability under a range of water regimes. Furthermore, there is need to investigate soil-root dynamics and extraction patterns of soil water at field levels by these species, when grown as a summer crop in rotation with winter wheat.

## Materials and methods

### Plant culture

The experiment was carried out in a greenhouse setting at the Oklahoma State University, Stillwater, OK (36.12° N, 97.06° W) during the spring season (March–May) of 2018. The greenhouse has 130 sq. m. space and is equipped with conventional heating and evaporating pad cooling systems. Seeds of tepary bean cv. PT082 (*Native Seeds*, Tucson, Arizona, USA), guar cv. Matador (*Guar Resources*, Brownfield, Texas, USA), and mothbean cv. PI426980 (*Plant Genetic Resources Conservation Unit*, Griffin, Georgia, USA) were sandwiched within moistened paper towels and kept at 28 °C for 2 days to induce germination. Six seeds showing radicle emergence were planted in polyvinylchloride pots (0.75 m tall, 0.15 diameter), which were filled with gravel at the bottom to permit flow of excess water through drainage holes, and the remainder of tubes were packed with pure, fine mason sand. Temperature inside the greenhouse was constantly set at 32/24 °C (day/night) during the experiment period. The applied photoperiod was extended to 14 h using supplemental lighting provided by a combination of metal halide and high-pressure sodium lamps. Temperature and humidity maintained inside the greenhouse was recorded every three minutes using a TP425 data logger (*The Dickson Company*, Addison, IL).

### Water deficit treatments

Plants of all three species were allowed to grow until 77 DAP. During the initial 4 weeks after planting, full-strength Hoagland nutrient solution^46^ was applied to every pot three times a day (0,800, 1,200, and 1,600 h) by an automated drip irrigation system that used a timing device to supply all plants with optimum water and nutrient conditions. The duration of irrigation events by the system was adjusted to maintain soil moisture near field capacity (0.10–0.11 m^3^ m^−3^)^47^ on regular basis, which was monitored by inserting TDR moisture probes (MiniTrase, Soilmoisture Equipment Corp., Santa Barbara, CA) to a depth of 15 cm in three reference pots for every species-water treatment combination. At 27 DAP, four different water treatments were randomly applied to thirty-two plants of each species, and continued until final harvest at 77 DAP (Fig. 1). Full-strength Hoagland nutrient solution was continuously used in each of four water treatments throughout the growing period. Among water treatments, the 100% treatment continued to receive full irrigation amount as a control, and three water deficit treatments: 75%, 50% and 25% of full irrigation, were assigned to other set of plants using timing devices (Fig. 1).

### Growth measurements

Four plants were randomly sampled to a 5-cm stubble height from each treatment combination at 48, 62, and 77 DAP. The number of leaves on each plant were counted, and leaf area was determined with an LI-3100 leaf meter (*LI-COR Inc*., Lincoln, NE). Each sampled plant was harvested and partitioned into leaves (including pods when encountered) and stems, and oven-dried at 65 °C to a constant weight, to determine dry weights of leaves and stems, and leaf-to-stem ratios. The sum of dry weights of leaves and stems was identified as total aboveground biomass. The rates of mainstem elongation, leaf addition, leaf growth, stem growth, leaf area expansion and biomass accumulation were estimated from length of mainstem, number of leaves, leaf weight, stem weight, leaf area and dry biomass weight, respectively, observed on the three sampling dates.

### Physiological measurements

Parameters for gas exchange and chlorophyll fluorescence were recorded using an LI-6400 photosynthesis system (*LI-COR Inc*., Lincoln, NE) on the third or fourth fully expanded leaves of plants between 11:00 and 13:00 h at 55 DAP. The instrument was set to the photosynthetic photon flux density of 1,200 µmol photon m^−2^ s^−1^, cuvette temperature of 32 °C, relative humidity of 35 ± 5%, and CO_2_ concentration of 400 µmol mol^−1^. Each measurement was logged when a steady-state was achieved. The quantum efficiency by oxidized open photosystem II reaction centers in light was determined as:

1 $$\frac{F{v}^{^{\prime}}}{F{m}^{^{\prime}}}=\frac{F{m}^{^{\prime}}-F{o}^{^{\prime}}}{F{m}^{^{\prime}}}$$

where Fm′ and Fo′ are maximum and minimum fluorescence, respectively, achieved in light-adapted leaves^48^. The *ETR* was calculated based on the equation^49^:

2 $$ETR=[(F{m}^{{\prime}}-Fs)/Fm{^{\prime}})] fI{\alpha }_{leaf}$$

where Fs is steady-state fluorescence, f is the fraction of absorbed quanta used by photosystem II, *I* is incident photon flux density, and α_leaf_ is leaf absorptance. *WUE*_*i*_ was estimated as the ratio of *A* and *T*. The ratio of *ETR/A* was used to estimate the number of electrons required to fix one CO_2_ molecule. After gas exchange recordings, the same plants were used for non-destructive chlorophyll measurements (*SPAD*) using a portable SPAD-502 chlorophyll meter (Konica Minolta Inc., Osaka, Japan).

### Statistical analysis

The experimental pots were arranged in a strip-plot design, with water treatments as the whole plots and legume species as strip plots. The relationship between *C*_i_/*C*_a_ and *g*_*s*_ was tested for linear, polynomial, and exponential function, and the best regression was selected based on coefficient of determination (r^2^) for each species. The relationships of *g*_*s*_ with ${{F_{v}^{{\prime }} } / {F_{m}^{{\prime }} }}$ and *ETR* were described by an exponential rise three-parameter regression function, [Y = y_0_ + (a * exp^bx^)]. An exponential decay three-parameter function, [Y = y_0_ + (a * exp^−bx^)], was used to describe relationship between *ETR/A* and *g*_*s*_. Rates of mainstem elongation, leaf addition, leaf growth, stem growth, leaf area expansion and biomass accumulation were calculated as the slope of a linear regression between observed values and days after the onset of water treatments. The relationship among the soil moisture content and estimated growth rates were fitted with second-order polynomial function, (Y = y_0_ + ax + bx^2^). All regression analyses were conducted using SigmaPlot version 14 (Systat Software Inc., San Jose, CA). Physiological parameters, involving *VpdL*, *A*, *g*_s_, *T*, *WUE*_*i*_ and *SPAD*, and growth parameters determined at final harvest were subjected to two-way analysis of variance (ANOVA) using PROC GLM in SAS 9.4^50^. After significant water x species interaction were noted at *p* ≤ 0.05, species treatments were tested at each individual water level using one-way ANOVA, and their means were separated using the Fisher’s least significant difference (LSD) comparison at *p* ≤ 0.05.

### Disclaimer

Mention of trademarks, proprietary products, or vendors does not constitute guarantee or warranty of products by USDA and does not imply its approval to the exclusion of other products that may be suitable. All programs and services of the USDA are offered on a nondiscriminatory basis, without regard to race, color, national origin, religion, sex, age, marital status, or handicap.
